# AKAP150-mediated TRPV1 sensitization is disrupted by calcium/calmodulin

**DOI:** 10.1186/1744-8069-7-34

**Published:** 2011-05-14

**Authors:** Sraboni Chaudhury, Manjot Bal, Sergei Belugin, Mark S Shapiro, Nathaniel A Jeske

**Affiliations:** 1 Department of Oral and Maxillofacial Surgery, University of Texas Health Science Center, San Antonio, TX, USA; 2 Department of Physiology, University of Texas Health Science Center, San Antonio, TX, USA; 3 Department of Endodontics, University of Texas Health Science Center, San Antonio, TX, USA; 4 Department of Pharmacology, University of Texas Health Science Center, San Antonio, TX, USA

## Abstract

**Background:**

The transient receptor potential vanilloid type1 (TRPV1) is expressed in nociceptive sensory neurons and is sensitive to phosphorylation. A-Kinase Anchoring Protein 79/150 (AKAP150) mediates phosphorylation of TRPV1 by Protein Kinases A and C, modulating channel activity. However, few studies have focused on the regulatory mechanisms that control AKAP150 association with TRPV1. In the present study, we identify a role for calcium/calmodulin in controlling AKAP150 association with, and sensitization of, TRPV1.

**Results:**

In trigeminal neurons, intracellular accumulation of calcium reduced AKAP150 association with TRPV1 in a manner sensitive to calmodulin antagonism. This was also observed in transfected Chinese hamster ovary (CHO) cells, providing a model for conducting molecular analysis of the association. In CHO cells, the deletion of the C-terminal calmodulin-binding site of TRPV1 resulted in greater association with AKAP150, and increased channel activity. Furthermore, the co-expression of wild-type calmodulin in CHOs significantly reduced TRPV1 association with AKAP150, as evidenced by total internal reflective fluorescence-fluorescence resonance energy transfer (TIRF-FRET) analysis and electrophysiology. Finally, dominant-negative calmodulin co-expression increased TRPV1 association with AKAP150 and increased basal and PKA-sensitized channel activity.

**Conclusions:**

the results from these studies indicate that calcium/calmodulin interferes with the association of AKAP150 with TRPV1, potentially extending resensitization of the channel.

## Background

The transient receptor potential vanilloid type1 (TRPV1) demonstrates a key role in injury and inflammatory conditions that can precipitate allodynia and hyperalgesia [1,2]. TRPV1 is a ligand-gated ion channel belonging to the transient receptor potential (TRP) family and is primarily expressed in peripheral c- and Aδ fibers [3]. TRPV1 participates in physical and chemical pain-evoked signal transduction, as it is activated by capsaicin, noxious heat (>42°C, [2]), low pH [4], cannabinoids including anandamide [5,6], and certain lipids [7]. TRPV1 contains multiple phosphorylation sites that are modified by protein kinase C (PKC) [8-10] and protein kinase A (PKA) [11-13] that play an important role in its sensitivity to agonist-directed activation. Additionally, TRPV1 interacts with a number of modulatory proteins including cytoskeleton proteins [14], the plasma membrane-associated protein Pirt [15] and the scaffolding protein A-kinase anchoring protein 79/150 (AKAP79 is the human ortholog, AKAP150 is the rodent ortholog) [16-19]. Importantly, AKAP150 modulates PKA- and PKC-mediated phosphorylation and mediates the activity of the TRPV1 receptor [16,17]. However, it is unclear whether certain signaling mechanisms mediate the association of TRPV1 with AKAP150.

TRPV1 is a cation-permeable channel whose activation results in Ca^2+ ^influx, resulting in membrane depolarization [2]. The rise in intracellular calcium stimulates several signaling cascades that can affect TRPV1 activity, including the calcineurin/protein phosphatase 2B (PP2B) pathway [20]. Following calcium-mediated activation of calcineurin, the phosphatase is capable of de-phosphorylating and de-sensitizing TRPV1 [21]. Indeed, both the chelation of extracellular calcium or co-treatment with calcineurin inhibitors reduce Ca^2+^-dependent desensitization of TRPV1 in cultured DRG neurons [22]. Previous studies on the amino acid sequence of TRPV1 have identified both N- and C-terminal sites capable of binding calmodulin [23-25], although the C-terminal binding site has demonstrated more significance in the tachyphylactic desensitization of TRPV1 [24]. Adjacent to the C-terminal binding site, Zhang et al. proposed a binding site for AKAP150, producing a 13-mer peptide corresponding to the TRPV1 C-terminal sequence (AA 736-749 of human TRPV1) capable of blocking association of the scaffolding protein and the receptor channel [19]. Considering the large size of AKAP150 (150 kDa), it is possible that a signaling process that associates with the C-terminal end of TRPV1 may occlude association of the receptor channel with AKAP150.

In the present study, we investigate the potential role that calcium/calmodulin has on AKAP150 association with TRPV1. Using biochemical, molecular, and imaging techniques, we sought to determine whether the primary mechanism that drives tachyphylactic desensitization of TRPV1, also actively dissociates AKAP150 from the receptor channel. Such a mechanism would provide a calcium-dependent regulatory process that preserves TRPV1 channel desensitization as to not over-activate nociceptive neurons.

## Results and Discussion

### The association of AKAP150 and TRPV1 in the plasma membrane is calcium sensitive

**Figure 1:**
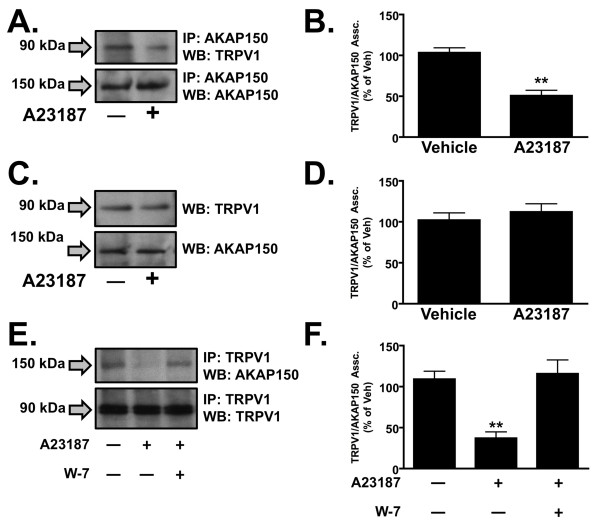
**TRPV1 association with AKAP150 is calcium sensitive**. (A) Co-immunoprecipitation and Western blot analysis of TRPV1 and AKAP150 following A23187 (1 μM, 10 min) in trigeminal neurons. (B) Quantified densitometry of data represented in (A), n = 4. (C) Plasma membrane expression of TRPV1 and AKAP150 does not change following A23187 exposure. (D) Quantified densitometry of data represented in (C), n = 3. (E) Co-immunoprecipitation of TRPV1 and AKAP150 from cultured TG neurons pretreated with A23187 and/or W-7 (calmodulin antagonist (100 μM; 30 min). (F) Quantified densitometry of data represented in (E), n = 5. Significance to vehicle treatment determined by two-tailed, paired t-test, **p < 0.01.

We have previously demonstrated AKAP150 association with TRPV1 in the plasma membrane of cultured trigeminal ganglia (TG, [16]. To determine the calcium-sensitivity of this association, we employed the calcium ionophore A23187 to induce calcium influx. In cultured TG neurons, A23187 treatment (1 μM, 10 min) decreased AKAP150 association with TRPV1 in the plasma membrane (Figure 1A-B), but did not effect plasma membrane-expression of either the scaffolding protein or the receptor channel (Figure 1C). These results suggest that a minor fraction of plasma-membrane localized AKAP150 associates with TRPV1, as there are more proteins that bind the scaffolding protein besides TRPV1 (for review, see [26]). The calcium-dependent signaling molecule calmodulin (CaM) mediates several post-translational events upon TRPV1 [24,27,28], prompting us to investigate whether this molecule is directing the calcium-sensitive association of AKAP150 and TRPV1. To discern this, we pre-treated cultured TG neurons with the CaM antagonist W-7 (100 μM, 30 min) prior to A23187 treatment, and observed a reversal of the calcium-dependent dissociative effect on AKAP150 and TRPV1 (Figure 1D). These data suggest that calcium-mediated dissociation of AKAP150 and TRPV1 involves the calcium-sensitive signaling protein CaM in cultured TG.

### Role of intracellular calcium in the association of AKAP150 and TPRV1

**Figure 2:**
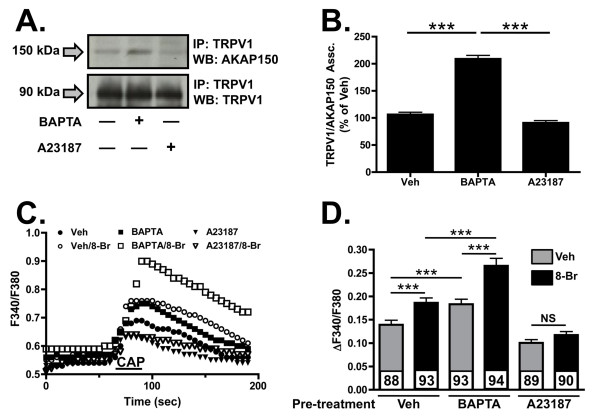
**Intracellular calcium effects TRPV1 and AKAP150 association and TRPV1 activity**. (A) Co-immunoprecipitation and Western blot analysis from CHO cells following BAPTA-AM (50 μm, 10 min) or A23187 (1 μm, 10 min). (B) Quantified densitometry of co-immunoprecipitation normalized to total TRPV1 expression. (C) Representative calcium-imaging traces of capsaicin (CAP)-stimulated (50 nm, 30 sec) intracellular Ca^2+ ^accumulation from CHO cells expressing TRPV1 and AKAP150. Cells pretreated with vehicle (H_2_0, veh), BAPTA-AM (50 μm, 10 min) or A23187 (1 μm,10 min) followed by 8-Br-cAMP (10 μm, 30 sec) administration. (D) Data represented in C are quantified as ΔF340/F380, mean ± SEM shown, n shown for each treatment paradigm. Significance determined by one-way ANOVA with Bonferroni correction, NS = no significance, *** p < 0.001.

We next assessed the role of calcium in the association of AKAP150 and TRPV1 in a transfected, serum-starved CHO cell model. Unlike TG neurons, A23187 treatment did not yield significant changes in AKAP150 association with TRPV1 in plasma membrane homogenates (Figure 2A-B). However, BAPTA-AM-driven chelation of intracellular calcium resulted in a significant increase in AKAP150 association with TRPV1, indicating that similar to cultured TG, intracellular calcium interferes with the association of the scaffolding protein and receptor channel in the plasma membrane compartment. Following Western blot analysis of co-immunoprecipitates, TRPV1 activity was monitored by calcium imaging, to determine whether calcium-directed changes in AKAP150 association with TRPV1 would effect capsaicin sensitivity of TRPV1. As shown in Figure 2C-D, BAPTA-AM pre-treatment not only increased basal TRPV1 activity, but also significantly increased PKA-mediated sensitization (8-Br-cAMP, 10 μM, 30 sec) of the receptor channel. Furthermore, A23187 pre-treatment of CHO cells reduced the significant effects of 8-Br-cAMP pre-treatment observed in vehicle-treated cells. Taken together with co-immunoprecipitation results, the functional association of AKAP150 with TRPV1 and its ability to direct PKA-mediated sensitization of TRPV1 [16,18,19] are sensitive to intracellular calcium.

### Role of calmodulin in AKAP150-mediated TRPV1 activity

**Figure 3:**
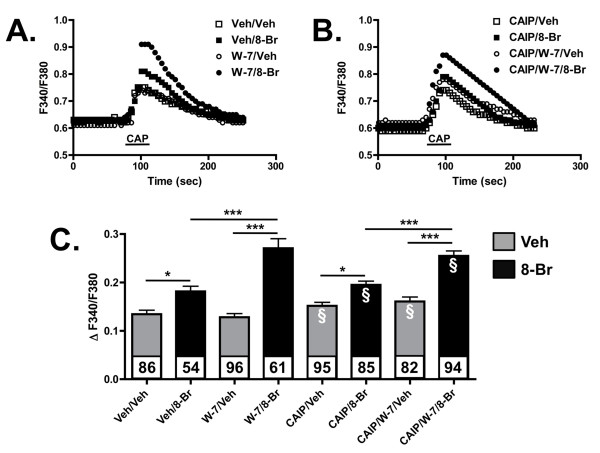
**Calmodulin inhibition increases AKAP150- mediated sensitization of TRPV1 activity**. (A-B) Representative traces of CAP-stimulated (50 nM, 30 sec) intracellular Ca^2+ ^accumulation from CHO cells expressing TRPV1 and AKAP150. Cells pretreated with vehicle (H_2_0, veh) or W-7 (100 μM) or CAIP (50 μM) for 30 min followed by 8-Br-cAMP (10 μm, 30 sec) administration. (C) Data represented in A are quantified as ΔF340/F380, mean ± SEM shown, n shown for each treatment paradigm. The white § indicates sample groups pre-treated with calcineurin auto-inhibitory peptide (CAIP). Significance determined by one-way ANOVA with Bonferroni correction, *p < 0.01, ***p < 0.001.

Previous studies have demonstrated that CaM modulates TRPV1 and can affect channel activity in Ca^2+^-dependent manner [24,25]. To determine the role of CaM in AKAP150-dependent PKA-sensitization of TRPV1 in serum-starved CHO cells, we pre-treated transfected cells with the CaM antagonist W-7 (100 μM, 30 min), followed by 8-Br-cAMP (10 μM, 30 sec), to determine the effects on TRPV1 activation by capsaicin (CAP, 50 nM, 30 sec) using calcium imaging. As reported previously [16,18,19], 8-Br-cAMP treatment sensitized the CAP-response in AKAP150 and TRPV1-expressing cells (Figure 3A, C). Importantly, W-7 pretreatment significantly increased the PKA-sensitized CAP-response in cells treated with 8-Br-cAMP, over those treated with vehicle and 8-Br-cAMP (Figure 3C). Next, we utilized a cell-permeable calcineurin autoinhibitory peptide (CAIP) to determine whether the effects of W-7 in this study were due to the subsequent inhibition of calcineurin-directed changes in TRPV1 activity. As demonstrated in Figure 3B-C, CAIP did not significantly effect any changes in TRPV1 activity induced by W-7-mediated inhibition of CaM. Importantly, CAIP also failed to significantly effect basal vehicle or 8-Br-cAMP-stimulated activity. Indeed, W-7 still significantly increased the PKA-sensitized CAP-response in cells treated with 8-Br-cAMP, over those treated with CAIP and 8-Br-cAMP. These results indicate that the effects of CaM inhibition on AKAP150 association with and subsequent modulation of TRPV1 are directly related to CaM.

### Deletion of calmodulin binding-sites on TRPV1 influences AKAP150 association with TRPV1

**Figure 4:**
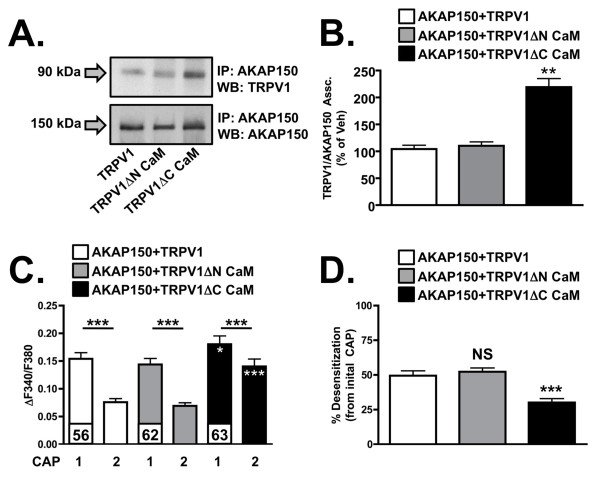
**Deletion of calmodulin-binding sites on TRPV1 effects TRPV1 and AKAP150 association**. (A) Co-immunoprecipitation and Western-blot analysis of AKAP150 with TRPV1, TRPV1ΔN CaM (TRPV1 mutant with deleted N-terminal calmodulin binding site) or TRPV1ΔC CaM (TRPV1 mutant with deleted C-terminal calmodulin binding site) in CHO cells. Results are representative of 4 independent trials. (B) Quantified densitometry of data represented in (A), n = 3. Significance with AKAP150 + TRPV1 transfection paradigm determined by determined by two-tailed, paired t-test, **p < 0.01. (C) Data from calcium-imaging traces of capsaicin (CAP)-stimulated (50 nM, 30 sec) intracellular Ca^2+ ^accumulation from CHO cells expressing AKAP150 and TRPV1, TRPV1ΔN CaM TRPV1ΔC CaM are quantified as ΔF340/F380, mean ± SEM shown, n shown for each treatment/transfection paradigm. White asterisks indicate significance between AKAP150 and TRPV1ΔCCaM expressing cells and AKAP150 and TRPV1 expressing cells. (D) Data in C are transformed to illustrate percent desensitization. Significance determined by one-way ANOVA with Bonferroni correction, NS = no significance, ***p < 0.001.

The TRPV1 amino acid sequence contains two confirmed CaM binding sites: one on the intracellular C-terminus [24] and one on the intracellular N-terminus [23]. To determine the relevance of CaM binding to TRPV1 as an inhibitor of AKAP150 association with the receptor channel, we mutationally deleted either the C-terminal or N-terminal CaM binding sequences in TRPV1 cDNA. Then, we compared the association of the mutants and full-length TRPV1 with AKAP150, and determined how the sequence deletions affect aspects of TRPV1 activity. As depicted in Figure 4A, deletion of the C-terminal CaM binding sequence (TRPV1 C CaM) resulted in greater co-immunoprecipitation of the mutant TRPV1 with AKAP150 over the N-terminal deletion mutant (TRPV1ΔN CaM) or full-length TRPV1. The fidelities of the mutations were confirmed by looking at their effects on the tachyphylactic desensitization of TRPV1 by repeated CAP-applications. The deletion of the C-terminal CaM binding site of TRPV1 significantly effected the tachyphylactic desensitization of TRPV1, as shown in Figure 4B-D, similar to other reported findings [24]. However, the effect of the C-terminal CaM binding site deletion could also be driven by the increased association of AKAP150 with the mutant TRPV1, thereby allowing for sensitization of the channel. This is evident in Figure 4C, as the basal CAP-response in TRPV1ΔC CaM was significantly higher than for full-length TRPV1 or TRPV1ΔN CaM. This also effected the percent desensitization reported in Figure 4D, as increased AKAP150 association with TRPV1ΔC CaM would drive increased CAP-response.

**Figure 5:**
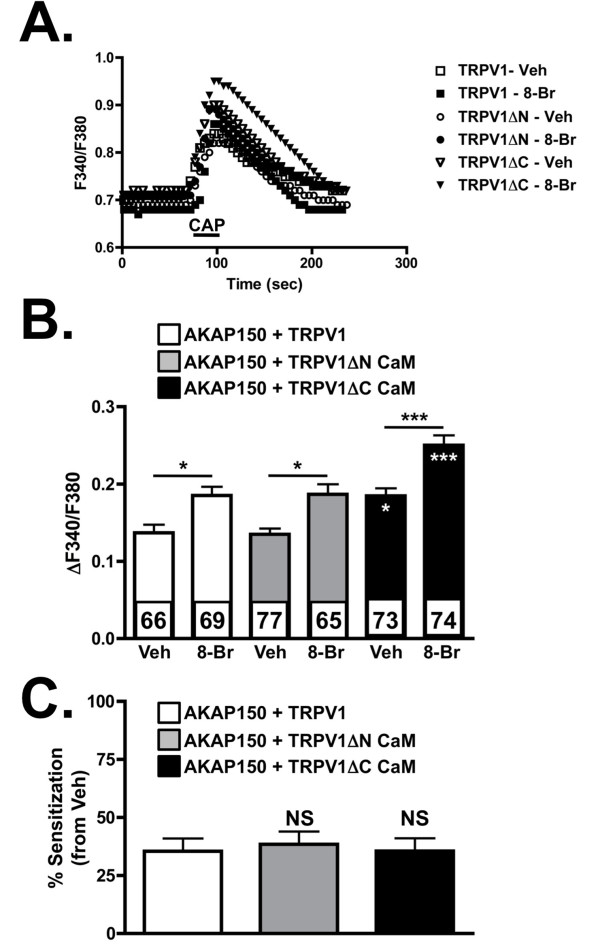
**Deletion of calmodulin-binding site on TRPV1 does not affect AKAP150-mediated TRPV1 activity**. (A) Representative calcium-imaging traces of CAP-stimulated (50 nM, 30 sec) intracellular Ca^2+ ^accumulation from CHO cells expressing AKAP150 and TRPV1, TRPV1ΔN CaM or TRPV1ΔC CaM following vehicle (Veh, H_2_0) or 8-Br-cAMP (8-Br-cAMP, 10 μM, 30 sec) pre-treatment. (B) Data represented in(A) are quantified as ΔF340/F380, mean ± SEM shown, n shown for each treatment/transfection paradigm. Black asterisks indicate significance between indicated groups. White asterisks indicate significance between marked treatment group and AKAP150 and TRPV1 expressing treatment groups (Veh-treated AKAP150 + TRPV1 ΔC CaM v. Veh-treated AKAP150 + TRPV1, for example). (C) Data in B are transformed to illustrate percent sensitization following 8-Br-cAMP pre-treatment. Significance determined by one-way ANOVA with Bonferroni correction, *p < 0.05, ***p < 0.001.

The deletion of the C-terminal CaM binding site of TRPV1 increased PKA-mediated CAP-responsiveness of TRPV1 in CHO cells co-expressing AKAP150 (Figure 5). Although there were significant changes in basal and 8-Br-cAMP-stimulated CAP-activity of TRPV1 (Figure 5B), there were no significant changes in percent sensitization from AKAP150 and full-length TRPV1 expressing cells (Figure 5C). These data suggest that deletion of the C-terminal CaM binding site of TRPV1 increases AKAP150 association.

### Calmodulin co-expression disrupts AKAP150 and TPRV1 association

**Figure 6:**
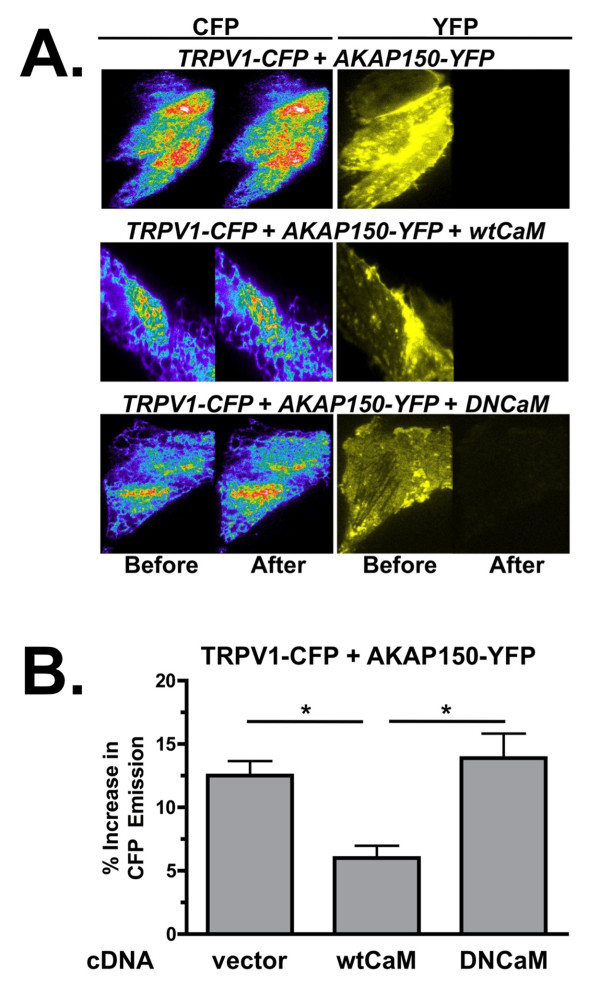
**Calmodulin co-expression decreases TRPV1 and AKAP150 TIRF/FRET signal**. (A) Images of CHO cells expressing CFP-tagged TRPV1 and YFP-tagged AKAP150 and either wild-type calmodulin (wtCaM) or dominant-negative calmodulin (DNCaM) under TIFR illumination, using 442 or 514 nm laser lines. Images of the CFP (in rainbow pseudocolor) and YFP (in yellow pseudocolor) emissions are shown before or after YFP photobleach, as labelled. (B) TIRF/FRET values from images acquired in A are quantified and illustrated as percent increase in CFP emission cells expressing the indicated calmodulin species. Vector = pcDNA3, mean ± SEM shown. Significance determined by Student's T-test, n = 9-12 cells/transfection paradigm, *p < 0.05.

**Figure 7:**
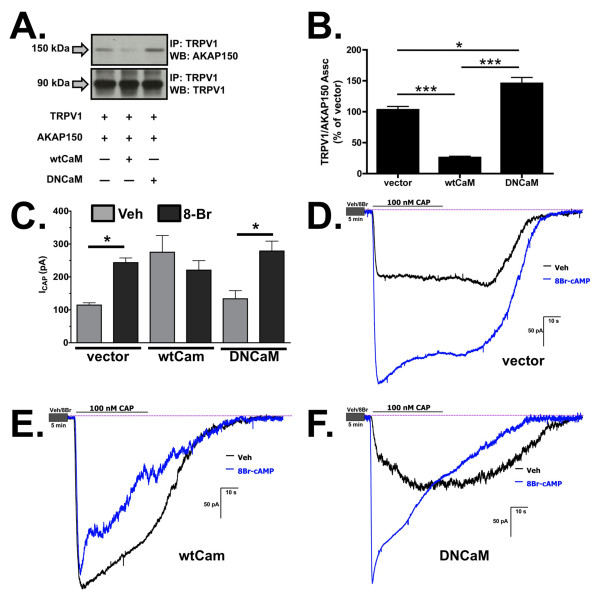
**Calmodulin co-expression decreases TRPV1 and AKAP150 association**. (A) Co-immunoprecipitation and Western blot analysis of TRPV1 and AKAP150 from CHO cells co-expressing wt CaM or DN CaM. (B) Quantified densitometry of co-immunoprecipitation results normalized to total TRPV1 expression. (C) CAP (100 nM, 30 sec) -current responses (100 nM, 30 sec) from CHO cells expressing AKAP150 and TRPV1 with empty vector pcDNA3, wt CaM, or DN CaM, following vehicle (Veh, H_2_0, gray bars) or 8-Br (8-Br-cAMP, 10 μm, 30 sec, black bars) pre-treatment, n = 6-9 per transfection/treatment paradigm. Black asterisks indicate significance between indicated groups. Significance determined by one-way ANOVA with Bonferroni correction, NS = no significance, *p < 0.05, ***p < 0.001. (D-F) Representative current traces from CHO cells expressing AKAP150, TRPV1, and empty vector (D), wtCaM (E), or DNCaM (F).

We next sought to determine whether the co-expression of calmodulin effects the association of AKAP150 and TRPV1 at the plasma membrane, using FRET measured by the donor-dequenching method under TIRF illumination. CHO cells were co-transfected with CFP-TRPV1, AKAP150-YFP and either wild type CaM (wtCaM) or a dominant-negative CaM (DNCaM, with all four Ca^2+^-binding sites mutated, preventing CaM from binding Ca^2+ ^[29-31]). In Figure 6, TIRF/FRET analysis demonstrates a significant decrease in the percentage of CFP emission in CHO cells over-expressing CFP-TRPV1, AKAP150-YFP, and wtCaM, versus those cells co-expressing empty vector (pcDNA3) or DNCaM (p < 0.05, one-way ANOVA, Bonferroni correction). The TIRF/FRET findings were similar to the effects that wtCaM and DNCaM had on AKAP150 association with TRPV1 by co-immunoprecipitation (Figure 7A-B). Significantly more AKAP150 associated with TRPV1 in the presence of DNCaM compared to cells co-transfected with empty vector, highlighting the inhibitory role that the calcium-signaling molecule plays in the association of AKAP150 and TRPV1. Whole-cell electrophysiology was utilized to determine the functional extent of AKAP150 association with TRPV1, using 8-Br-cAMP treatment to stimulate PKA-directed sensitization of TRPV1, which is AKAP150-dependent [13,16,18,19]. In Figure 7C-F, the co-expression of wtCaM with TRPV1 and AKAP150 inhibited PKA-mediated sensitization of TRPV1 following 8-Br-cAMP pre-treatment, suggesting reduced AKAP150 association with the receptor channel. In contrast, DNCaM co-expression allowed PKA-mediated sensitization of TRPV1. Thus, the presence of CaM disrupts AKAP150 and TRPV1 association and any subsequent AKAP150-mediated TRPV1 sensitization by PKA.

## Conclusions

The calcium sensor and signal transducer CaM, serves multiple roles in a variety of cell types, often translocating from one cellular compartment to another in response to increases in intracellular calcium [32]. In the case of TRPV1, CaM serves to mediate calcium-dependent events that include calcineurin-dependent de-phosphorylation and desensitization of TRPV1 [24,25], as well as CaM Kinase II-dependent phosphorylation and sensitization of the receptor channel [27,28]. In this study, we sought to determine the role of calcium and/or CaM in mediating AKAP150 association with TRPV1 in both cultured primary afferent neurons and immortalized CHO cells. In cultured TG, calcium influx inhibited AKAP150 association with TRPV1, in a manner sensitive to pharmacological inhibition of CaM. Furthermore, CaM inhibition increased PKA-directed sensitization of TRPV1, a process known to be dependent upon functional AKAP150 association [13,16,18,19]. Experiments that tested the effects of CaM inhibition on TRPV1 activity in the presence of calcineurin inhibitors suggest that the CaM-associated protein calcineurin does not contribute to the reported results. Finally, the co-expression of wtCaM or DNCaM with AKAP150 and TRPV1 in the CHO model yielded results that indicate that CaM alone is sufficient to reduce functional association of the scaffolding protein and receptor channel, using TIRF/FRET analysis, co-immunoprecipitation, and electrophysiology. In light of the general assumption that CaM plays a major role in the desensitization of TRPV1, it may play a larger role in preventing the resensitization of TRPV1 by blocking AKAP150 association and subsequent phopshorylation of the receptor channel.

In experimental results presented here, it is assumed that CaM primarily associates with TRPV1. In fact, AKAP79, the human ortholog to AKAP150, has demonstrated an ability to also bind CaM in environments of high calcium [33]. PKC and CaM compete for a similar binding site on AKAP79, such that when intracellular calcium increases and CaM is present, AKAP79 binds the calcium/CaM complex and "releases" PKC from neuronal post-synaptic densities to interact with other proteins and participate in adjacent signaling events [33]. The data presented here circumvent this theory, in that it has been previously demonstrated that functional expression of AKAP150 is required for PKC-mediated phosphorylation and sensitization of TRPV1 [17,19]. Furthermore, the introduction of CaM via heterologous expression in AKAP150- and TRPV1-expressing CHO cells resulted in a significant reduction in TRPV1 activity. If CaM association with AKAP150 were significant in this scheme, one would expect to observe increased CAP-responses following CaM co-expression, since TRPV1 activity is sensitive to PKC-mediated phosphorylation [8,10]. However, the results presented herein specifically demonstrate significantly reduced TRPV1 activities in the presence of CaM, indicating that the calcium-sensitive molecule interferes with the association of AKAP150 and TRPV1.

The deletion of CaM binding sites in the TRPV1 sequence provided interesting findings in our studies, in the context of previously published studies using similar TRPV1 mutants. Numazaki et al (2003) identified a 35-amino acid C-terminal sequence of TRPV1 responsible for binding CaM following rises in intracellular calcium. The corresponding amino acids in the rat cDNA were deleted (TRPV1ΔC CaM) for studies presented here. We also created a TRPV1 mutant missing the N-terminal CaM binding site (TRPV1ΔN CaM), based on previous reports [23,25]. In agreement with findings reported by Rosenbaum et al. (2004), we demonstrate that the presence of calcium/CaM significantly reduces TRPV1 activity, in a manner that indicates channel desensitization. Furthermore, we were able to demonstrate a significant effect of CaM-inhibition on AKAP150 association with TRPV1 and subsequent CAP-responses. However, others have been unable to demonstrate that W-7 treatment can significantly block CAP-directed tachyphylactic desensitization [20,34], thereby suggesting that CaM may not associate with TRPV1 as a free molecule, but rather as an associative part of a calcium-sensitive complex.

The CaM binding sites of TRPV1 are required for multiple signaling mechanisms that ensure channel activity, nociceptor viability, and overall health and protection of the organism. In this study, we identify CaM association with TRPV1 as a mechanism that prevents AKAP150 association with and subsequent sensitization of the receptor channel. Following the activation of TRPV1 by a nociceptive stimulus, the influx of calcium ions act to bind and activate CaM, which then associates with TRPV1 [24,25]. The CaM complex formation with TRPV1 effectively prohibits AKAP150 from associating with TRPV1. It is this mechanism that is postulated to prolong desensitization of the channel, as AKAP150 association with TRPV1 would allow for recovery from its de-phosphorylated, desensitized state. The results presented here may help to contribute to understanding the various molecular mechanisms that contribute to the specific states of TRPV1 channel desensitization following tachyphylaxis.

## Methods

### Tissue Culture

The Institutional Animal Care and Use Committee of UTHSCSA has approved all procedures for the use of animals, which were conducted in accordance with policies for the ethical treatment of animals established by the National Institutes of Health. Trigeminal ganglia (TG) were dissected bilaterally from male Sprague-Dawley rats (200-250 g; Charles River Laboratories, Wilmington, MA) and dissociated by treatment with collagenase (Lot# S5K8219, Worthington, Lakewood, NJ) for 30 min, followed by treatment with trypsin (Sigma, St. Louis, MO) for 15 min. Cells were centrifuged, aspirated and re-suspended in Dulbecco's modified Eagle's medium (Invitrogen, Carlsbad, CA) supplemented with 10% fetal bovine serum (Invitrogen), 100 ng/ml nerve growth factor (Harlan Laboratories, Indianapolis, IN), 1% penicillin/streptomycin (Invitrogen) and 1% glutamine (Sigma), and then placed on poly-D-lysine coated plates. Cultures were maintained at 37°C and 5% CO_2 _and grown for 5-7 days. Chinese hamster ovarian (CHO) cells were utilized for heterologous expression of cDNA constructs. CHO cells were maintained in Dulbecco's modified Eagle's medium (Cellgro, Mediatech Inc. Manassas, VA) with 10% fetal bovine serum (Invitrogen, Carlsbad, CA) along with 1% penicillin (Invitrogen) and streptomycin (Invitrogen) at 37°C and 5% CO_2_. CHO ells were transiently transfected with equal amounts of each cDNA per experiment, using Lipofectamine 2000 (Invitrogen) following the manufacturer's instructions.

### Crude plasma membrane preparation, Immunoprecipitation and Western Blot Analysis

CHO cells and cultured TG were harvested as previously described [21]. W-7 (100 μM, 30 min treatment, Sigma Aldrich), BAPTA-AM (1,2-bis(o-aminophenoxy)ethane-N, N, N', N'-tetraacetic acid tetrakis(acetoxymethyl ester); 50 μM, 10 min treatment, Sigma Aldrich), CAIP (calcineurin autoinhibitory peptide, [21], 100 μM, 30 min treatment, EMD Chemicals, Inc., Gibbstown, NJ) or A23187 (1 μM,10 min treatment, Sigma Aldrich) was used to treat the cells at 37°C prior to cell harvest. Following homogenization by 20 strokes in a Potter-Elvehjem homogenizer in a hypotonic homogenization buffer (25 mM HEPES, 25 mM sucrose, 1.5 mM MgCl2, 50 mM NaCl, pH 7.2), the whole-cell homogenate was incubated on ice for 15 min and then centrifuged at 1000 g for 1 min at 4°C to remove nuclei and unlysed cells from the homogenate. The supernatant was isolated and centrifuged at 16 000 g for 30 min at 4°C, separating cytosolic proteins from cell membrane proteins. The pellet (crude plasma membrane fraction) was then re-suspended in 500 μL homogenization buffer containing 1% Triton X-100 (Fisher Scientific, Pittsburgh, PA). The protein contents of crude plasma membrane fractions were quantified using the Bradford assay [35] as recommended by the manufacturer (Sigma). Following protein quantification, crude plasma membrane fractions (200 μg) were incubated with 1 μg of anti-AKAP150 (R-300, Santa Cruz Biotechnology, Santa Cruz, CA) or anti-TRPV1 (R-130, Santa Cruz Biotechnology) for 2 hr at 4°C. Samples were then centrifuged at 16 0000 g for 1 min at 4°C to remove unsolubilized material, and the supernatant was incubated with protein-A agarose beads (Sigma) for 1 hr at 4°C, followed by 4 washes of the beads with homogenization buffer containing 1% Triton. Immunoprecipitates were resolved via 12.5% SDS-polyacrylamide and transferred to polyvinyl difluoride membrane (Millipore, Billerica, MA) for immunoblotting. Western blots were blocked in 5% non-fat milk in Tris-buffered saline/Tween 20 and incubated in anti-AKAP150 or anti-TRPV1, followed by the appropriate horseradish peroxidase-conjugated secondary antisera (GE Healthcare, Piscataway, NJ). Enhanced chemiluminescence (GE Healthcare) was used following manufacturer's instructions for visualization of antigen-antibody binding.

### Generation of CaM-binding site TRPV1 mutants

Rat TRPV1 cDNA (generously provided by David Julius) was used for cloning of TRPV1ΔNCaM and TRPV1ΔCCaM in pECFP-C1 vector. For both TRPV1ΔNCaM and TRPV1ΔCCaM, the mutations were performed using restriction enzymes Spe I site and EcoR V sites, respectively. The forward (F) and reverse (R) primers used for generating mutants TRPV1ΔNCaM were identified as F- 5'-TTTACTAGTAATGGAGCAGATGTCCAGGC-3' and R- 5'-AAAACTAGTCTGCTTCAGGCTGTCTGC-3' and TRPV1ΔCCaM, F- 5'-TTTGATATC CGAGATAGACATGCCACC-3' and R- 5'-AAAGATATCACAGTTGCCTGGGTCCTCG-3'. All mutants were generated by Quickchange mutagenesis (Stratagene, La Jolla, CA), and all clones were verified by DNA sequencing.

### Calcium Imaging

CHO cells were transfected as outlined above (Tissue Culture) with pEGFP-N1 (Clontech, Mountain View, CA, to identify transfected cells) and cDNA vectors containing inserts corresponding to rat TRPV1 (generously provided by David Julius, UCSF, San Francisco, CA), rat AKAP150 wt (generously provided by John D. Scott, University of Washington, Seattle, WA), and TRPV1 ΔN CaM and TRPV1 ΔC CaM. To measure intracellular [Ca^+2^] levels, the dye Fura-2 AM (2 μM; Molecular Probes, Carlsbad, CA) was loaded for 30 min at 37°C into cells in the presence of 0.05% Pluronic (Calbiochem). Fluorescence was detected with a Nikon Eclipse TE 2000-U microscope fitted with a 20×/0.8 NA Fluor objective. Fluorescence images from 340 nm and 380 nm excitation wavelengths were collected and analyzed with the MetaFluor Software (MetaMorph, Web Universal Imaging Corporation, Downingtown, PA). To assess for Ca^+2 ^accumulation following TRPV1 activation, capsaicin (CAP, 50 nM; Sigma Aldrich) was administered for 30 sec followed by a 3 min wash with standard extracellular solution buffer (SES, 140 mM NaCl, 5 mM KCl, 2 mM CaCl_2_, 1 mM MgCl_2_, 10 mM D-(+)-glucose, 10 mM HEPES, Ph 7.4). The net change in Ca^+2 ^(ΔF340/380) was calculated by subtracting the basal F340/380 Ca^+2 ^level (mean value collected for 60 s prior to CAP addition) from the peak F340/380 Ca^+2 ^level achieved after exposure to the CAP. When required, the PKA activator 8-Br-cAMP (10 μM) was applied to the cell for 30 sec prior to CAP administration. For each transfection/treatment group, 54-96 cells were imaged, statistical significance determined by one-way ANOVA analysis, with Bonferroni correction as needed. Percent desensitization determined by calculating the percent difference of the second CAP response from the first for each recorded trace.

### Total internal reflection fluorescence microscopy (TIRF) microscopy

Fluorescence emissions from enhanced cyan fluorescent protein (CFP)-tagged AKAP150 or enhanced yellow fluorescent protein (YFP)-tagged TRPV1 were collected at room temperature using TIRF microscopy [36]. All TIRF experiments were performed in the total internal reflection fluorescence microscopy core facility housed within the Department of Physiology, UTHSCSA. Fluorescence emissions were collected using an inverted Nikon TE2000 microscope with through-the-lens (prismless) TIRF imaging (Nikon). This system is equipped with a vibration isolation system (Technical Manufacturing, Peabody, MA) to minimize drift and noise. Samples were viewed through a plan-Apo TIRF 60× oil-immersion high resolution (1.45 numerical aperture) TIRF objective. Coupled to the microscope is a laser light delivery system (Prairie Technologies, Middleton, WI) consisting of a 40 mW argon laser outputting 488 and 514 nm lines and a 442 nm diode pumped solid-state laser. The excitation light was selected with an acoustic optical tunable filter, controlled by MetaMorph software. CFP and YFP emissions were simultaneously collected using the Dual-View chip splitter (Optical Insights), equipped with a filter cube containing HQ470 nm/30 m and HQ550 nm/30 m emission filters for CFP and YFP emission, respectively, and a 505 nm dichroic mirror for separation of emission wavelengths. In this configuration, the microscope uses only a dual-bandpass TIRF dichroic mirror to separate the excitation and emission light, with no excitation filters in the microscope cube. The TIRF angle was adjusted by eye to give the signature TIRF illumination to the experimental chamber. Fluorescence images were collected and processed with a 16 bit, cooled charge-coupled device camera (Cascade 512F; Roper Scientific, Tucson, AZ) interfaced to a PC running MetaMorph software. This camera uses a front-illuminated EMCCD with on-chip multiplication gain. Images were collected (200-600 ms exposure time, adjusted to best exploit the dynamic range of the camera without pixel saturation) immediately before and after photobleaching. Images were not binned or filtered, with pixel size corresponding to a square of 122 × 122 nm.

### Fluorescence resonance energy transfer (FRET)

We used either the "acceptor photobleaching" (donor dequenching) or "sensitized emission" method of evaluating FRET efficiency. In the former, the emission of the donor fluorophore is compared before and after photobleaching of the acceptor [37]. YFP photobleaching was performed using the 100 W mercury lamp of the microscope, using a standard YFP filter cube. We find that 5-7 min excitation by the mercury lamp using the YFP cube is sufficient to photobleach >75% of the YFP fluorophores, yet results in negligible photobleaching of the CFP fluorophores. The following protocol was used: the medium in the glass-bottomed dishes was exchanged with Ringer's solution that contained the following (in mM): 160 NaCl, 5 KCl, 1 MgCl_2_, 2 mM CaCl_2_, and 10 HEPES, pH 7.4 with NaOH. Cells were first examined using the mercury lamp and standard CFP or YFP filter cubes to find a suitable cell robustly expressing both CFP- and YFP-tagged proteins. Under TIRF illumination, the focal plane used is critical, and was adjusted if necessary immediately before each image acquisition to obtain a sharp TIRF image. The focusing and cell-centering protocol resulted in CFP photobleaching of <1%. TIRF images using 442 and 514 laser lines were acquired before and after photobleaching of the YFP fluorophores. %FRET was calculated as the percentage increase in CFP emission after YFP photobleaching by using the following formula: percent FRET = [(CFPpost - CFPpre)/CFPpre] × 100, where CFPpost is CFP emission after YFP photobleaching, and CFPpre is CFP emission before YFP photobleaching. The percent FRET was calculated by drawing regions of interest around the entire area of the cell and subtracting the background in a cell-free region for each image.

For measurement of TIRF/FRET using the "sensitized emission" method, the sample was excited at 442 nm, and CFP and YFP emission simultaneously collected using the Dual-View/camera combination, where the split sides of the camera chip will be referred to as the CFP and the YFP channels, respectively. To quantify the effective FRET efficiency for each experiment, we use the "3-cube" method used by many investigators [38-41], based on the formalism of Gordon et al. [42]. Our system does not use three physical "cubes," but rather the Dual-View; however, the principle, which we outline here, is the same. To quantify the "bleed-through" of the CFP emission into the YFP channel, cells expressing only ECFP-M were excited at 442 nm, and the image intensities of the cell on the CFP and YFP channels quantified, yielding a ratio of YFP channel/CFP channel of 26%. By exciting cells only expressing EYFP-M, we determined that the bleed-through of YFP emission into the CFP channel is zero, as is the excitation of CFP by 514 nm light. The direct excitation of YFP at 442 nm is calculated as a ratio of YFP excitation at 514 nm, and this was quantified using cells only expressing EYFP-M, yielding a value of 4.3%. Thus, to measure sensitized-emission FRET, the cells are sequentially imaged under 442 and 514 nm laser lines under TIRF. The intensity of the cell region in the YFP channel is first scaled down by 26% of the intensity of the identical cell region in the CFP channel, to isolate the YFP emission. The total YFP emission is then broken down into that arising from direct excitation by the 442 nm light, and that from energy transfer from CFP. The former is calculated by multiplying the 514 nm image in the YFP channel by 4.3%, with the remainder thus assumed due to FRET. We then calculate the ratio of total YFP emission to that from direct excitation of YFP, the "FRET ratio" (FR). The FR value is easily converted to FRET efficiency (Eeff) using the following equation: Eeff = (FR - 1) × (YFP,442/CFP,442), where YFP,442 and CFP,442 are the molar extinction coefficients for YFP and CFP when excited at 442 nm. From their published maximal molar extinction coefficients and known excitation spectra of YFP and CFP [43], the YFP,442/CFP,442 ratio was calculated to be 0.101, which is used to convert the FR to FRET efficiency.

### Electrophysiology

Recordings were made in whole-cell perforated patch voltage clamp (holding potential (V_h_) of -60 mV) configuration at 22-24°C from transfected CHO cells. Data were acquired and analyzed using an Axopatch 200B amplifier and pCLAMP 10.0 software (Molecular Devices). Recording data were filtered at 0.5 kHz and sampled at 2 kHz. Access resistance (R_s_) was compensated (40-80%) when appropriate up to the value of 13-18 MW. Data were rejected when R_s _changed >20% during recording, leak currents were >50pA, or input resistance was <200 MW. Currents were considered positive when their amplitudes were 5-fold bigger than displayed noise (in root mean square). Standard external solution (SES) contained (in mM): 140 NaCl, 5 KCl, 2 CaCl_2_, 1 MgCl_2_, 10 D-glucose and 10 HEPES, pH 7.4. The pipette solution for the perforated patch configurations consisted of (in mM): 140 KCl, 1 MgCl_2_, 10 HEPES pH 7.3 and 250 mg/ml amphotericin B (Sigma, St. Louis, MO). Drugs were applied using a fast, pressure-driven and computer controlled 8-channel system (ValveLink8; AutoMate Scientific, San Francisco, CA).

## Abbreviations

AKAP150: A-kinase anchoring protein 150; BAPTA-AM: 1,2-bis(o-aminophenoxy)ethane-N: N: N': N'-tetraacetic acid tetrakis(acetoxymethyl ester); CAIP: calcineurin autoinhibitory peptide; CaM: calmodulin; AP: capsaicin; CHO: Chinese hamster ovary; FRET: Fluorescence resonance energy transfer; PKA: protein kinase A; PKC: protein kinase C; TIRF: total internal reflection fluorescence; TRPV1: transient receptor potential vanilloid receptor type 1

## Competing interests

The authors declare that they have no competing interests.

## Authors' contributions

SC, MB, and SB performed experiments. SC, MS and NJ analyzed data. SC, MS, and NAJ wrote the manuscript. All authors read and approved the final manuscript.
